# Predicting distribution of *Aedes aegypti* and *Culex pipiens* complex, potential vectors of Rift Valley fever virus in relation to disease epidemics in East Africa

**DOI:** 10.3402/iee.v3i0.21748

**Published:** 2013-10-14

**Authors:** Clement Nyamunura Mweya, Sharadhuli Iddi Kimera, John Bukombe Kija, Leonard E. G. Mboera

**Affiliations:** 1 National Institute for Medical Research, Tukuyu Research Centre, Tukuyu, Tanzania; 2 Department of Veterinary Medicine and Public Health, Sokoine University of Agriculture, Morogoro, Tanzania; 3 Serengeti Biodiversity Programme, Tanzania Wildlife Research Institute, Arusha, Tanzania; 4 National Institute for Medical Research, Dar es Salaam, Tanzania

**Keywords:** Rift Valley fever, predicting distribution, Maxent, Aedes aegypti, Culex pipiens, East Africa

## Abstract

**Background:**

The East African region has experienced several Rift Valley fever (RVF) outbreaks since the 1930s. The objective of this study was to identify distributions of potential disease vectors in relation to disease epidemics. Understanding disease vector potential distributions is a major concern for disease transmission dynamics.

**Methods:**

Diverse ecological niche modelling techniques have been developed for this purpose: we present a maximum entropy (Maxent) approach for estimating distributions of potential RVF vectors in un-sampled areas in East Africa. We modelled the distribution of two species of mosquitoes (*Aedes aegypti* and *Culex pipiens* complex) responsible for potential maintenance and amplification of the virus, respectively. Predicted distributions of environmentally suitable areas in East Africa were based on the presence-only occurrence data derived from our entomological study in Ngorongoro District in northern Tanzania.

**Results:**

Our model predicted potential suitable areas with high success rates of 90.9% for *A. aegypti* and 91.6% for *C. pipiens* complex. Model performance was statistically significantly better than random for both species. Most suitable sites for the two vectors were predicted in central and northwestern Tanzania with previous disease epidemics. Other important risk areas include western Lake Victoria, northern parts of Lake Malawi, and the Rift Valley region of Kenya.

**Conclusion:**

Findings from this study show distributions of vectors had biological and epidemiological significance in relation to disease outbreak hotspots, and hence provide guidance for the selection of sampling areas for RVF vectors during inter-epidemic periods.

Rift Valley fever (RVF) is a mosquito-borne arboviral infection of zoonotic importance with major socio-economic implications (1). RVF virus is passed from generation to generation of aedine mosquitoes trans-ovarially, accounting for the continued presence of the virus in enzootic foci (2–4). Outbreaks have been reported across much of sub-Saharan Africa, North Africa, Saudi Arabia, and Madagascar (5, 6). In East Africa, regular epidemics of RVF disease have been reported since the 1930s (7). Most socio-economic severe outbreaks in Tanzania, Kenya, and Somalia were in 1997–1998 and 2006–2007 (8, 9); human deaths were 478 in 1998 and 309 in 2007 (10). The 2007 outbreak was the most widespread affecting livestock in 11 regions in Tanzania and Kenya (11, 12). A total of 16,973 cattle, 20,193 goats, and 12,124 sheep died of the disease, with spontaneous abortions reported in 15,726 cattle, 19,199 goats, and 11,085 sheep (13, 14).

Vertical transmission in aedine mosquitoes provides the virus with a sustainable mechanism of persistence as eggs that can survive for several years in dry conditions during inter-epidemic periods (2, 15–17). Emergence of infected mosquito populations and amplification of the virus are apparently determined by changes in climate and weather conditions (15, 18, 19). Important RVF vectors in East Africa include *Aedes mcintoshi, A. ochraeus, Culex pipiens, A. dalzieli*, and *A. vexans* (20). Despite records that indicate *A. mcintoshi* as the main vector for RVF in Kenya (21–23), *Aedes aegypti* has been found naturally infected with RVF virus in Sudan in 2007, and it has been demonstrated in the laboratory to be capable of transmitting the virus both mechanically and biologically (24). Laboratory-established colonies of *A. aegypti* from Tahiti exhibited the highest disseminated infection rates of RVF virus when compared with other potential vectors in the Mediterranean (25). *A. aegypti* has also demonstrated infection and transmission rates of the nonstructural proteins (NSs) deletion virus similar to wild-type virus, while dissemination rates were significantly reduced (26). *C. pipiens* was incriminated as the main RVF vector in Egypt based on field isolates and laboratory experiments (27). Moreover, publications have shown that populations of *C. pipiens* from the Maghreb and South Africa are efficient experimental vectors of RVF (28, 29).

Predicting potential RVF vector distributions is useful in providing more understanding about the disease ecology. It can guide planning future disease control and prevention interventions, such as strategic animal vaccination and vector control (30). Maxent, similar to other ecological niche models (ENM) use occurrence data with environmental data layers to estimate the species’ potential occurrence in the study area (31). ENM algorithms aim to predict environmental suitability for the species as a function of the given environmental variables (32). Maxent has been used to estimate the probability distribution for the suitability of conditions for a species’ occurrence based on environmental constraints (31).

Published examples indicate that Maxent performs well in characterising suitability for species, even with small sample sizes (33–36) particularly, when well-designed survey data and functionally relevant predictors are analysed with an appropriately specified model. ENM tools have been used widely in many ecological applications (31, 37, 38). It is envisaged that this study will add to understanding distributions of RVF vectors in East Africa and guiding vector sampling during inter-epidemic periods.

## Material and methods

### Source of species occurrence data

**Fig. 1:**
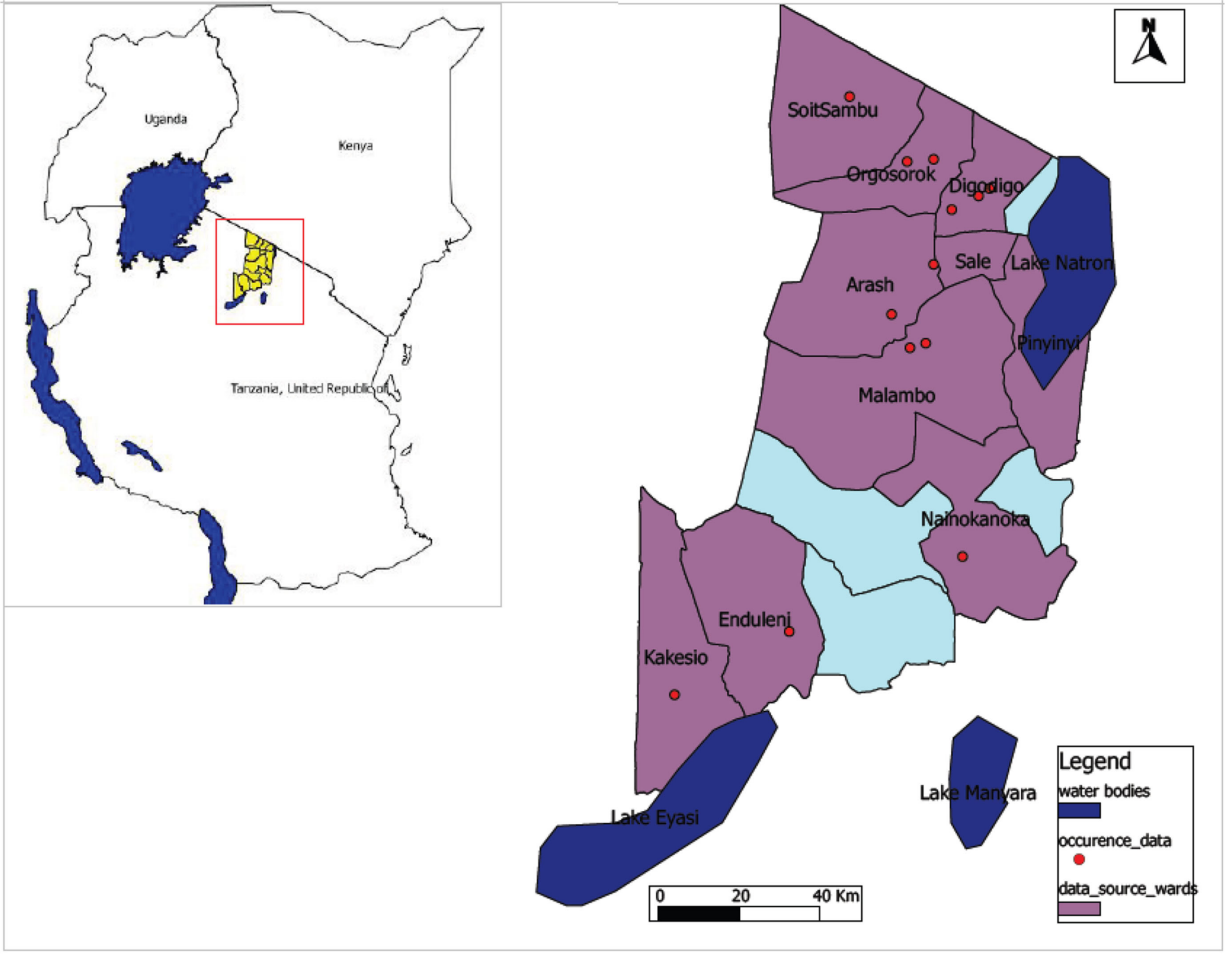
Map of Ngorongoro District indicating wards as sampling areas for *A. aegypti* and *C. pipiens* complex occurrence data.

Occurrence data were derived from our entomological fieldwork in Ngorongoro District in northern Tanzania (Fig. 1) as RVF epidemic hotspots. Outdoor and indoor mosquito collections were made using CDC light traps baited with CO_2_ sachets and Mosquito Magnets (Mosquito Magnet Cordless Liberty Plus) baited with Octenol attractants (39). Adult mosquitoes were identified morphologically using specific keys (40). Traps set in each site were geo-referenced. Ngorongoro District was chosen as a source of species occurrence data due to the history of RVF outbreak in Tanzania linked with Kenya (10). According to the 2012 Tanzania National population and housing census, the population at risk of infection was 174,278 (41). The District is considered as part of the Serengeti-Mara Ecosystem, which is defined by the limits of the annual wildlife migration. Our Ngorongoro District study area represents a unique zone of interaction between livestock, wildlife, and humans with animal migration from a nearby country.

### Modelling procedure

Maxent was used to investigate potential distributions of RVF vectors across East Africa. The method estimates distributions at maximum entropy subject to a set of constraints that represent existing information about the target distribution (32). Maxent employs species occurrence data and environmental variable layers for the study area (31). Modelling potential distribution of RVF vectors, for each species, 13 random partitions of the occurrence localities were made. Each partition was created by randomly selecting 70% of the occurrence localities for training, with the remaining 30% reserved for testing (33, 35).

### Environmental variables

Environmental variables were obtained from the World-Clim dataset (http://www.worldclim.org/bioclim.htm). All variables were tested, but only eight bioclimatic and one topographic variable were used as predictors for *A. aegypti* and *C. pipiens* complex distributions. Variables were chosen based on their relevance to mosquito vector distributions after several jackknifing procedures as described by Pearson et al. (33). Variable contributions to our models were determined initially by iteration of the algorithm; the change in regularised gain with and without the corresponding variable is assessed. For a second estimate, for each environmental variable in turn, the values of that variable on training presence and background data are randomly permuted; the model is re-evaluated on the permuted data, and the resulting drop in gain is assessed.

### Model evaluation

A jackknifing approach was employed to assess model predictive performance (33). Our choice for this approach to model evaluation was based on a relatively small number of available occurrence records with no absence data to characterise commission errors. Many previous models used receiver operating characteristic (ROC) approaches, which require both absence and presence data (31), and which present numerous other problems (42). In light of lack of absence data, jackknifing provides the best option as it does not require absence data. Twelve different predictions were run for each species: during each run, one of the occurrence records was excluded. The excluded record was then checked to see if the model was able to include it in the predicted suitable area. We used the *p*-value compute programme provided as supplementary material to Pearson et al. (33) to test the significance of the model. ROC and area under the curve (AUC) procedure was also applied to our model output for comparison (31).

## Results

**Fig. 2:**
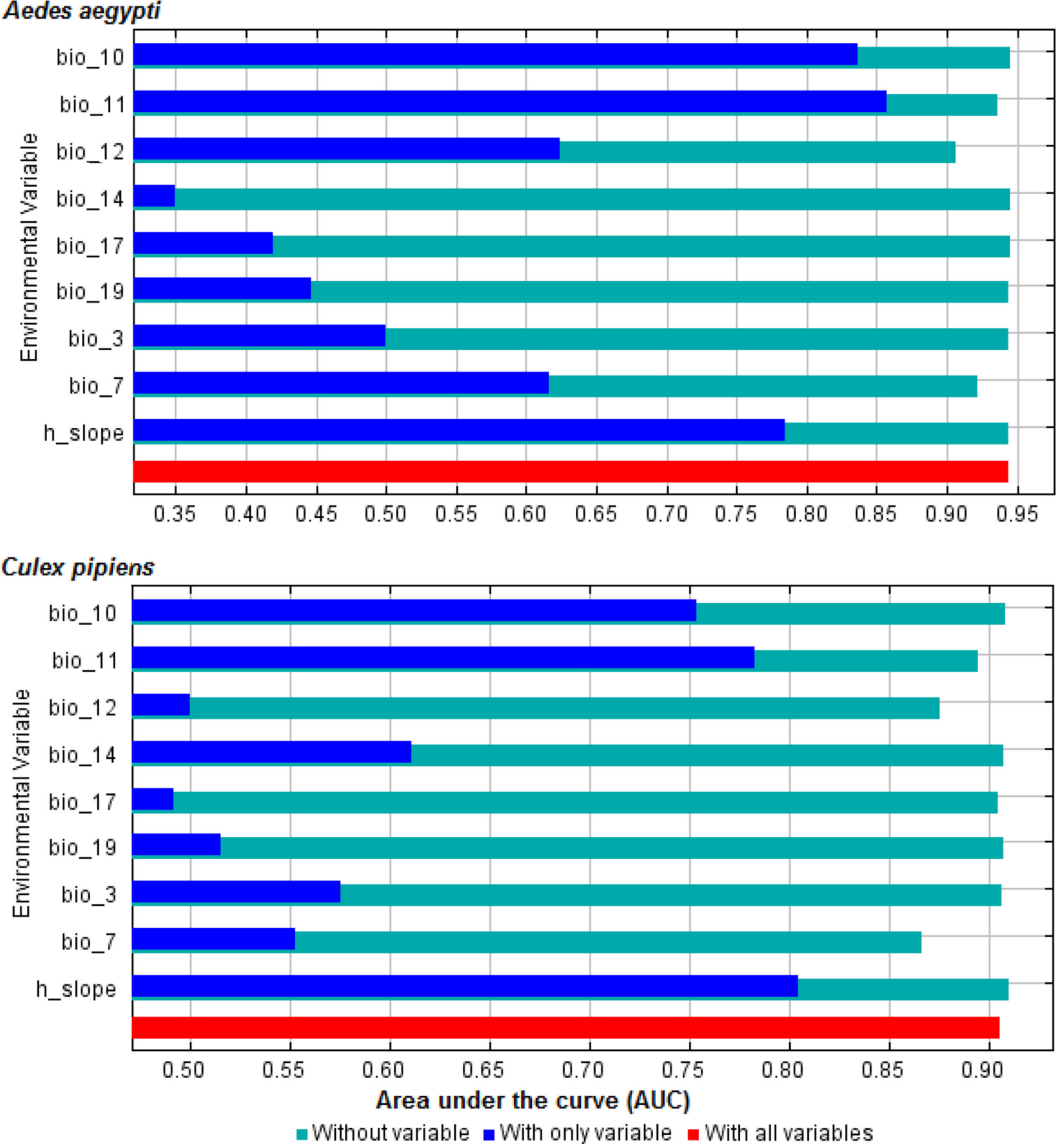
Results of jackknife of area under the curve indicating relative importance of predictor variables: red bar shows model performance will all variables, blue bar show model performance with each variable alone, and light sky blue colour shows model performance without each variable.

The selection of environmental predictor variables showed that mean temperature of coldest quarter, temperature annual range, mean temperature of warmest quarter, precipitation of coldest quarter, annual precipitation, precipitation of driest quarter, isothermality, slope, and precipitation of driest month had significant contributions to model quality (Fig. 2). The jackknife test of variable importance showed that ‘mean temperature of coldest quarter’ and temperature annual range presented the highest gain and thus were the two most important predictors of *A. aegypti* and *C. pipiens* complex distributions.

The predicted suitability maps show that the probability of the presence of *A. aegypti* and *C. pipiens* complex appears to be moderate to high over large areas of East Africa. Our model predicted potential suitable areas with high success rates of 90.9% for *A. aegypti* and 91.7% for *C. pipiens* complex. Model performance was statistically significantly better than random expectations for both species (*p*<0.0001). Similarly, ROC/AUC values were 0.945 for *A. aegypti* and 0.908 for *C. pipiens* complex. Subjectively, AUC scores above 0.9 indicate excellent model predictive performance (43). Probability of suitability was medium to high in Rift Valley area experiencing RVF outbreaks.

**Fig. 3:**
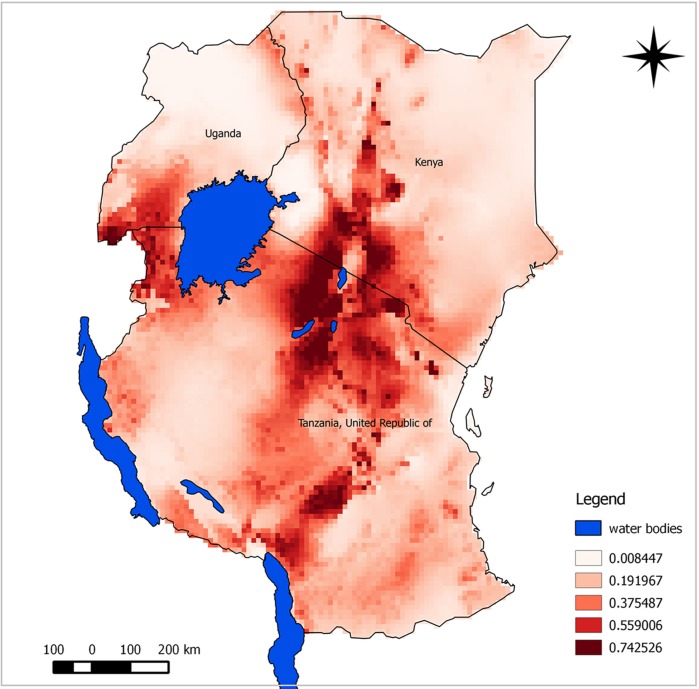
Predicted potential suitable distributions area for *A. aegypti* in East Africa associated with RVF outbreak history; the image uses colours to indicate predicted suitability.

**Fig. 4:**
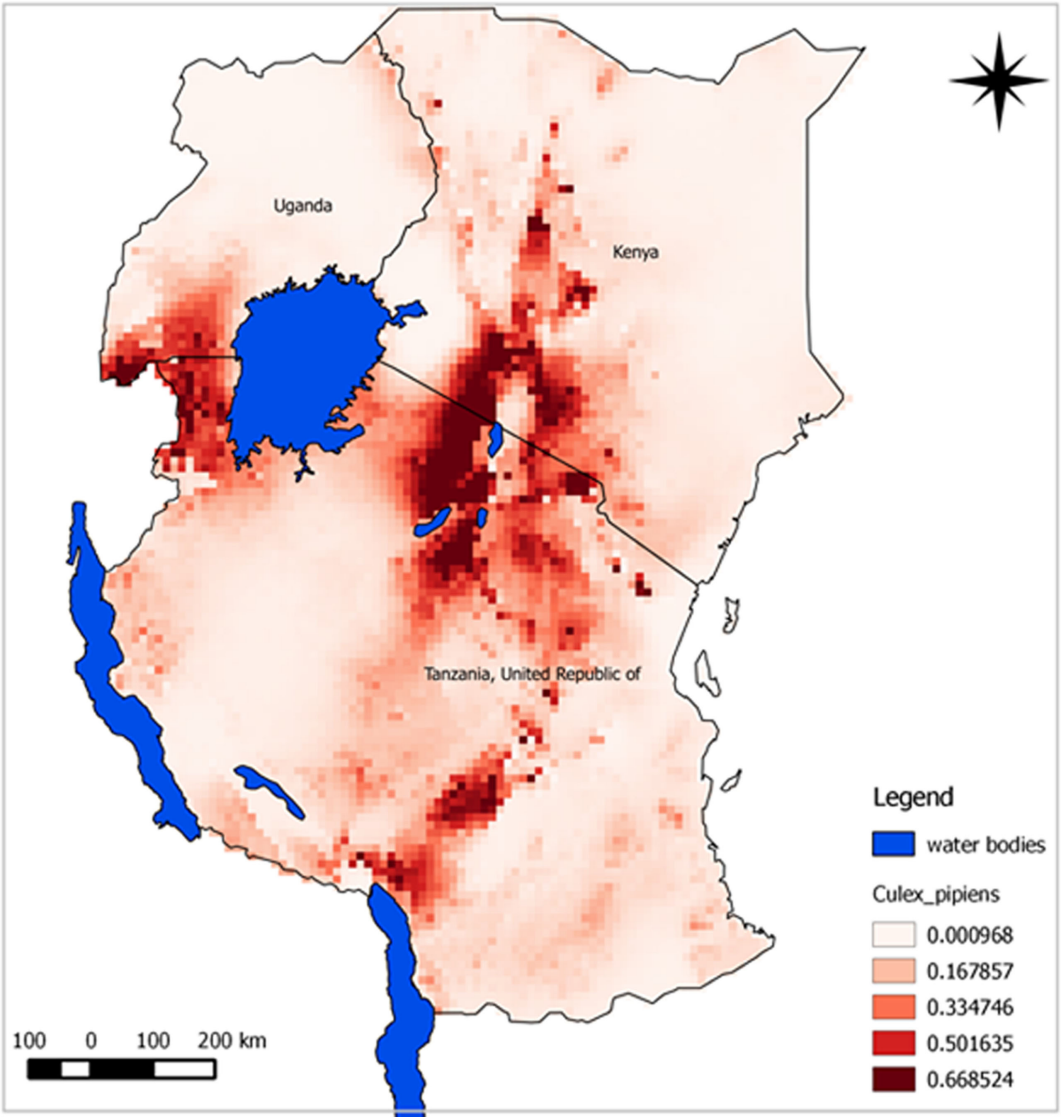
Predicted potential suitable distributions area for *C. pipiens* complex in East Africa associated with RVF outbreak history; the image uses colours to indicate predicted suitability.

The predicted maps presented in Figs. 3 and 4 identify suitable areas for sampling for RVF vectors as follow-up for better understanding of disease transmission dynamics. Most areas shown in the map with high risk have also indicated as areas where previous RVF outbreaks have been recorded. Most suitable sites for the two vectors were in the central and north-west areas of Tanzania. Other important risk areas include western areas of Lake Victoria, northern Lake Malawi, and the Rift Valley region of Kenya.

## Discussion

In this study, our model was highly successful in identifying potential distribution patterns of vectors, which appear to show intriguing associations with disease epidemic records. Hence distribution patterns for RVF vectors use a small number of occurrence records and environmental variables using ecological niche modelling. We therefore provide the first model-based distribution maps for *A. aegypti* and *C. pipiens* complex in East Africa. Our maps show that distribution areas for *A. aegypti* and *C. pipiens* complex correspond closely to the Great Rift Valley region where RVF outbreaks have occurred. However, other parts also have been identified as being suitable including west of Lake Victoria. More research is needed to determine whether these areas have RVF vectors.

Information produced during this study is timely and highly relevant, given the threats of RVF outbreaks in parts of Tanzania and Kenya due to increased rainfall trends over the past several months as this paper was prepared. Also climate trends may result in conditions that favour the emergence of disease. Therefore, mapping potential risk areas for RVF vectors can help in planning disease control strategies and discovering previously unknown risk areas, and also to identify top-priority sites for animal vaccination against RVF virus towards control of disease epidemics.

The methodology presented here could also be used for quantifying potential distribution patterns for other disease vectors in East Africa, such as the *Anopheles gambiae* complex and may aid field surveys and allocation of disease control. Therefore, these results are especially pertinent towards guiding public health workers across the East African countries because they identify areas most susceptible to colonisation, allowing them to focus on control efforts.

However, we suggest that the predicted presence of *A. aegypti* and *C. pipiens* complex should be interpreted with caution especially in the southern parts of Uganda, as model sensitivity may decline as the predicted suitable areas were based on only a few data points of mosquitoes collected in Ngorongoro District, Tanzania. Further research is required to identify more RVF vectors in other areas with records of disease epidemics; inclusion of disease vectors such as *A. mcintoshi* may enable the capture of suitable distribution of the RVF vectors in other areas such as eastern Kenya.

In conclusion, predicted distribution maps are initial steps in understanding the transmission of RVF virus during inter-epidemic periods in East Africa. Our ecological niche modelling approach was able to arrogate occurrences of mosquitoes as risk maps of disease outbreaks. Species distribution maps can help to inform researchers and disease control teams targeting areas for mosquito sampling and surveillance. This predicted distribution suggests a more comprehensive assessment of the risk implied to distribution of these vectors for targeted disease control strategies such as vector control and animal vaccination. Our hope is that this will provide a useful tool for further research on ecological factors associated with disease distributions in East Africa.
